# The association between cytokines and psychomotor speed in a spectrum of psychotic disorders: A longitudinal study

**DOI:** 10.1016/j.bbih.2021.100392

**Published:** 2021-11-23

**Authors:** Jeanette Brun Larsen, Solveig Klæbo Reitan, Else-Marie Løberg, Maria Rettenbacher, Øystein Bruserud, Tor Ketil Larsen, Liss Anda, Christoffer Bartz-Johannessen, Erik Johnsen, Rune A. Kroken

**Affiliations:** a Department of Mental Health, St. Olav's University Hospital, Trondheim, Norway; b Department of Mental Health, Faculty of Medicine and Health Science, Norwegian University of Science and Technology, Trondheim, Norway; c NORMENT, Division of Psychiatry, Haukeland University Hospital, Bergen, Norway; d Department of Addiction Medicine, Haukeland University Hospital, Bergen, Norway; e Department of Clinical Psychology, University of Bergen, Bergen, Norway; f Department of Psychiatry, Psychotherapy and Psychosomatics, Medical University Innsbruck, Innsbruck, Austria; g Department of Clinical Science, University of Bergen, Bergen, Norway; h Department of Medicine, Haukeland University Hospital, Bergen, Norway; i Department of Clinical Medicine, University of Bergen, Bergen, Norway; j Regional Centre for Clinical Research in Psychosis, TIPS, Stavanger University Hospital, Bergen, Norway; k Department of Social Studies, University of Stavanger, Stavanger, Norway

**Keywords:** Cytokines, Immune markers, Inflammation, Schizophrenia, Psychomotor performance, Psychomotor speed, Cognition, hs-CRP, high-sensitivity C-reactive protein, IL, interleukin, MRI, magnetic resonance imaging, MS, multiple sclerosis, PANSS, Positive and Negative Syndrome Scale, RCT, randomised controlled trial, SCID-I, Structured Clinical Interview for DSM-IV Axis I Disorders, TMT, Trail Making Test, TNF, tumor necrosis factor, BMI, body mass index

## Abstract

**Background:**

In schizophrenia, impaired psychomotor speed is a common symptom predicting worse functional outcome. Inflammation causes changes in white matter integrity, which may lead to reduced psychomotor speed. Therefore, we wanted to investigate if peripheral inflammation assessed with cytokines affected performance on psychomotor speed in patients with a spectrum of psychotic disorders.

**Methods:**

The current study is a prospective cohort study, including participants from a pragmatic, randomised controlled trial comparing three atypical antipsychotics in patients with a spectrum of psychotic disorders. For the purposes of this sub-study, we analysed drug treatment groups collectively. Psychomotor speed was assessed at baseline, and at weeks 6, 12, 26 and 52 of follow-up, using the neuropsychological tests trail making test (TMT) A and B, and symbol coding. Serum concentration of the following cytokines were measured: interleukin (IL)-β, IL-2, IL-4, IL-6, IL-10, IL12 p70, IL-17a, interferon (IFN)-γ and tumor necrosis factor (TNF)-α. Blood samples were collected at baseline and after 1, 3, 6, 12, 26, 39 and 52 weeks. We analysed the effect of cytokines levels on psychomotor speed over time in linear mixed effects models.

**Results:**

In our linear mixed effects models controlling for possible confounders, IFN-γ had a significant negative effect on TMT-A and symbol coding performance. None of the other tests for psychomotor speed were significantly associated with cytokines. Overall psychomotor speed performance increased significantly across the study period while cytokine levels remained stable.

**Conclusion:**

Our study indicates a negative association between IFN-γ and psychomotor speed, which might be of importance when understanding the mechanisms behind psychomotor deviations in psychotic disorders.

## Highlights

• The cytokine interferon (IFN) – γ is related to psychomotor speed in patients with psychotic disorders.
• For majority of cytokines, we found no significant association with psychomotor speed.
• Cytokines remained stable during the study period of 52 weeks.

## 1 Introduction

Deviations in psychomotor speed have been demonstrated in patients with various mental disorders, including schizophrenia and major depression (Bruder et al., 2014; Dickinson et al., 2007; Rybakowski and Borkowska, 2002). In patients with schizophrenia, psychomotor speed impairment is associated with worse functional and social outcome (Nuechterlein et al., 2011; Sanchez et al., 2009). Further, subjects at clinical high risk for psychosis show impairment in several cognitive domains, including psychomotor speed (Anda et al., 2019; Zheng et al., 2018). Interestingly, psychomotor speed may be a strong cognitive predictor for whether a subject in a potential prodromal state actually do develop psychosis (Pukrop et al., 2007).

In general, psychomotor speed is associated with reduced white matter integrity in magnetic resonance imaging (MRI) studies of healthy volunteers and patients with schizophrenia (Karbasforoushan et al., 2015; Wang et al., 2020). This relation is prominent in several areas of the brain, including the basal ganglia (Tsapanou et al., 2019). Inflammation is one pathway that might alter both white matter integrity and connectivity of the basal ganglia (Felger et al., 2016; Najjar and Pearlman, 2015), subsequently affecting psychomotor speed (Felger et al., 2016; Walker et al., 2017). Theoretically, this is possible knowing that peripheral cytokines can cross the blood-brain barrier both ways (Banks, 2015), bind to glial cells (Prieto and Cotman, 2017), and induce damage to oligodendrocytes and microglial cells (Martino et al., 2000). Additionally, inflammation and cytokines might influence dopamine levels in the basal ganglia (Petrulli et al., 2017), influencing performance on tests for psychomotor speed (Capuron et al., 2012).

The possible link between psychomotor speed and inflammation is of particular interest when studying patients with psychosis. There is increasing evidence for a linkage between psychosis and inflammation (Kroken et al., 2018; Pape et al., 2019). Notably, patients with schizophrenia have higher levels of inflammatory markers including cytokines, compared to healthy controls (Goldsmith et al., 2016b; Miller et al., 2011). Changes in cytokine levels across the course of illness is seen across a range of mental health disorders (including schizophrenia), with a common pattern where cytokine levels fluctuate between acute and chronic states (Goldsmith et al., 2016b; Momtazmanesh et al., 2019). There is a need for studies on inflammation in relation to signs and transdiagnostic symptoms (Khandaker et al., 2017). Psychomotor speed impairment may be one such transdiagnostic sign, evident in several mental disorders.

A few previous studies on psychotic disorders identified an association between reduced psychomotor speed and cytokines such as interleukin (IL) -6 and tumor necrosis factor (TNF) -α (Frydecka et al., 2015; Goldsmith et al., 2020). However, the results are conflicting as other studies found no significant association (Hori et al., 2017; Martínez-Cengotitabengoa et al., 2012). Moreover, previous studies are limited by cross sectional designs, which preclude conclusions about causality. Nor can cross sectional studies take into account that both cytokines and psychomotor speed might change over time. Therefore, we wanted to explore the association between cytokines and psychomotor speed in a longitudinal study including patients with spectrum of psychotic disorders. The aim was to investigate whether cytokine levels affected psychomotor speed over time.

## 2 Methods

### 2.1 Setting and participants

The current study is a prospective cohort study with participants from the BeStInTro study, a pragmatic, randomised controlled trial (RCT) comparing three different atypical antipsychotics in the treatment of schizophrenia spectrum disorders. The primary outcome results from this study are recently published in Lancet Psychiatry (Johnsen et al., 2020). The follow-up period was 52 weeks with visits at baseline as well as after 1, 3, 6, 12, 26, 39 and 52 weeks. Blood samples were collected at all these visits. Participants underwent neuropsychological testing at baseline, and at week 6, 12, 26 and 52. For the analyses of the present study, the patients were not subdivided into treatment groups.

Three treatment centers in Norway and one in Austria participated in collecting the data between Oct 20, 2011, to Dec 30, 2016. Inclusion criteria were age ≥18 years and a psychosis spectrum disorder (ICD-10 diagnosis F20-29) with active psychotic symptoms. Active psychosis was defined as a score ≥4 on at least one of the following Positive and Negative Syndrome Scale (PANSS) items: Delusions (P1), Hallucinatory behavior (P3), Grandiosity (P5), Suspiciousness/persecution (P6) or Unusual thought content (G9) (Kay et al., 1987). Exclusion criteria were inability to understand the site language, pregnancy, breastfeeding, limbic encephalitis, hypersensitivity to any of three antipsychotic drugs tested in the main study, or somatic disorders known as precautions for the drugs tested (prolactin-dependent tumors, pheochromocytoma, risk of torsade de points or narrow-angle glaucoma). The study protocol and population are previously described in detail elsewhere (Johnsen et al., 2020).

All patients gave written informed consent prior to inclusion. The study was approved by the Regional Committees for Medical and Health Research Ethics and the Norwegian Medicines Agency in Norway, and the Ethikkommission der Medizinische Universität Innsbruck and the Austrian Federal Office for Safety in Health Care in Austria. This work was carried out in accordance with the Declaration of Helsinki. The study is registered at ClinicalTrials.gov (NCT01446328).

### 2.2 Clinical assessments

Diagnoses were set according to the Structured Clinical Interview for DSM-IV Axis I Disorders (SCID-I) and converted to ICD-10 diagnoses in line with Norwegian clinical guidelines. Sociodemographic data were recorded at baseline. We assessed alcohol and drug abuse with the two scales the Clinical Alcohol Use Scale and Clinical Drug Use Scale (Drake et al., 1990). At baseline, we recorded information on demographic variables such as ethnicity, smoking status, gender and age. In addition, we interviewed participants with PANSS at each visit. Height at baseline, and weight were also measured throughout the entire study period in order to calculate body mass index (BMI, kg/m^2^). Participants underwent testing with Trail Making Tests A (TMT-A) and B (TMT-B) (Bowie and Harvey, 2006; Crowe, 1998), at baseline and at weeks 6, 12, 26 and 52. Symbol coding was conducted at baseline and weeks 6, 26 and 52 (Keefe et al., 2008). All raw scores from the cognitive tests were converted to t-scores according to available norms and manuals.

### 2.3 Immune biomarkers

Fasting-state blood samples were collected between 8 and 10 a.m. Before centrifugation at 3300 ​rpm for 10 ​min, the blood was left to clot at room temperature for 20–120 ​min. The aliquoted serum was frozen at -40 ​°C and stored at -80 ​°C. Cytokine analyses were conducted with a Multiplex immunoassay (High Sensitivity 9-Plex Human ProcartaPlex™ Panel (ThermoFisher Scientific, Waltham, MA, USA)). The following cytokines were analysed: IL-1β, IL-2, IL-4, IL-6, IL-10, IL-12 p70, IL-17a, interferon (IFN)–γ and TNF-α. The panel was performed in accordance with the manufacturers' instructions, but we substituted the universal assay buffer with 1X phosphate buffer saline (PBS) with 0.10% tween in order to improve fluorescence intensities. Using the manufacturers’ recommendations for sample dilutions and standard curve concentrations, all samples and standards were assayed in duplicates. A Luminex 200 (R&D Systems, Inc., Minneapolis, MN, USA) was used for measuring the fluorescence intensities, and two lots of reagents with similar upper and lower detection limits (ULOQ/LLOQ) were used for all samples (21 plates).

The detection limit was defined as the lowest detected value within the standard curve. Samples below the detection limits were set to half of the detection limits. Summarizing all visits, the number of samples and percentage under the detection limit was as follows: IL-1β: 229 (30.1%), IL-2: 41 (5.4%), IL-4: 162 (21.3%), IL-6: 239 (31.4%), IL-10: 74 (9.7%), IL-12 p70: 171 (26.3%), IL-17a: 40 (6.1%), IFN-γ: 158 (20.8%) and TNF-α: 146 (19.2%). Mean inter- and intra-assay coefficients of variation (CV) are given in Table A1.

### 2.4 Statistical analyses

Statistical analyses were performed using R version 3.6.2 for Windows (www.R-project.org). The level of significance was set at p ​≤ ​0.05 and all analyses were two-tailed. We evaluated data normality with Shapiro-Wilk normality test and evaluation of the QQ-plots. TMT-A, TMT-B and symbol coding t-scores were close to normally distributed. All cytokine values were heavily right skewed, and thus log transformed cytokine values were used in the statistical analyses. We assessed associations between demographic variables, cytokines, and tests for psychomotor speed with Pearson's correlation for continuous variables, and with Mann Whitney *U* test or *t*-test (depending on the normal distribution) for dichotomous variables.

We used linear mixed effects models to see if log transformed cytokines, TMT-A, TMT-B, or symbol coding changed over time alone without adjusting for the effect of each other. A random intercept for each patient was included to account for dependencies in the data due to repeated measures from the same participants. Secondly, we calculated the Pearson correlation coefficient for the relationship between log transformed cytokines, and TMT-A, TMT-B, or symbol coding at baseline and at end point. Finally, we used linear mixed effects models to assess the relationship between log transformed cytokines and tests for psychomotor speed. We made one model for each psychomotor test, including the test score as dependent variable, and cytokines and visit as independent variables. Again, a random intercept for each patient was included in order to account for dependencies in the data. The following possible confounders were included in the model as independent variables: age, gender, BMI, ethnicity, smoking, study site, antipsychotic drug and PANSS positive subscale score.

Due to the possible confounding effect of including patients with various psychotic disorders, we conducted sensitivity analyses where patients with F22 Delusional disorder and F23 Acute and transient psychosis were excluded. After excluding these patients, we conducted the same linear mixed effect models investigating the association between log transformed cytokines and tests for psychomotor speed correcting for possible confounders.

## 3 Results

The total sample consisted of 144 patients. At baseline, serum cytokine measurements were available for 140 patients, TMT-A scores for 101 patients, TMT-B scores for 89 patients and symbol coding scores for 92 patients. At end point, there were cytokine measurements from 58 patients, TMT-A scores for 47 patients, TMT-B scores for 40 patients and symbol coding scores for 41 patients. A total of 79 patients had measurements of cytokines and all three psychomotor tests at baseline, and 39 patients had both at end point. Table 1, Table 2 show demographic and clinical parameters at baseline.

**Table 1:** Demographic and clinical parameters at baseline (N ​= ​144).

	N	%	
Gender (female)	51	35.4	
Higher education (above high school)^a^	25	19.0	
Employed^b^	36	26.5	
Smokers^c^	84	66.1	
Alcohol misuse or dependence^a^	13	9.6	
Drug misuse or dependence^b^	27	19.9	
Diagnosis			
F20 Schizophrenia	84	58.0	
F21 Schizotypal disorder	2	1.4	
F22 Delusional disorders	21	14.6	
F23 Acute and transient psychosis	18	12.5	
F25 Schizoaffective	10	6.9	
F28–F29 Other and unspecified psychosis	9	6.3	
	**Mean**	**SD**	**Range**
Age	31.7	12.7	18–65
BMI^d^	25.5	6.0	16.4–54.8
PANSS total score	77.6	15.3	44.0–142.0
PANSS positive score	21.1	4.9	12.0–38.0
TMT-A (sec)	35.5	22.5	12–181
TMT-B (sec)	91.9	53.8	32–335
Symbol coding (t-score)	35.8	12.9	12–76

Abbreviations: BMI; body mass index, PANSS; Positive and Negative Syndrome Scale, TMT; Trail Making Test.

a Missing 9.

b Missing 8.

c Missing 17.

d Missing 22.

**Table 2:** Cytokine levels at baseline.

	Mean	SD	Range
IFN-γ (pg/ml)	5.3	9.4	0.04–70.87
IL-1β (pg/ml)	1.2	2.8	0.01–22.74
IL-10 (pg/ml)	1.6	2.5	0.02–22.10
IL-12 p70 (pg/ml)	3.2	8.2	0.12–80.82
IL-17a (pg/ml)	33.3	46.6	0.02–279.43
IL-2 (pg/ml)	25.5	31.9	0.06–217.47
IL-4 (pg/ml)	21.2	34.9	0.10–227.41
IL-6 (pg/ml)	2.0	3.1	0.07–15.19
TNF-α (pg/ml)	46.1	76.1	0.11–445.08

Abbreviations: IFN; interferon, IL; interleukin, TNF; tumor necrosis factor.

### 3.1 Change in psychomotor speed and cytokines over time

**Fig. 1:**
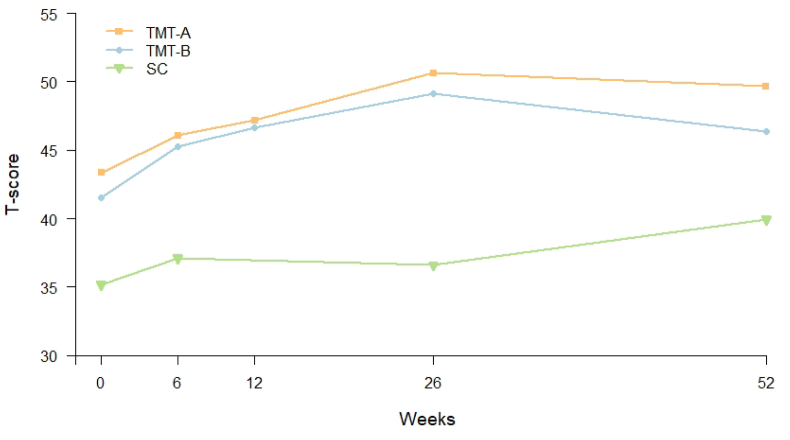
Estimated mean t-score of psychomotor speed over time. Data estimated in linear mixed effect models including three different tests for psychomotor speed. Abbreviations: TMT; trail making test, SC; symbol coding.

**Fig. 2:**
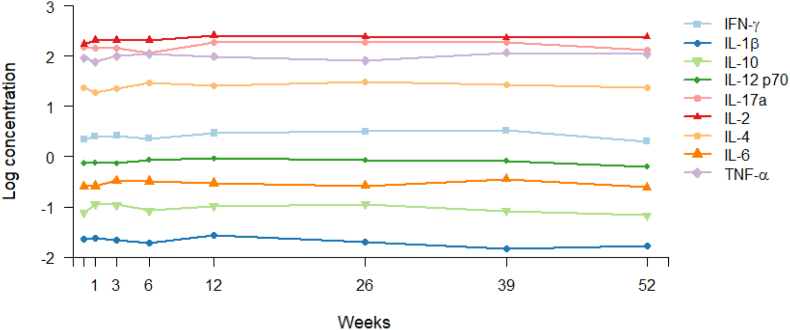
Estimated mean levels of cytokines over time. Data estimated in linear mixed effect models including log transformed cytokine values. Abbreviations: IFN; interferon, IL; interleukin, TNF; tumor necrosis factor.

Overall, the mean level of t-score for the tests TMT-A, TMT-B and symbol coding increased during the study period in linear mixed effects models, being most pronounced for TMT-A (Fig. 1). From baseline to end point, there were significant changes in t-scores for TMT-A (mean change ​= ​6.3, p-value < 0.001), TMT-B (mean change ​= ​4.9, p-value < 0.001) and symbol coding (mean change ​= ​4.8, p-value < 0.001). Further, a linear mixed effects model found a statistically significant improvement on performance for the TMT-A t-score from baseline to 6 weeks (mean change ​= ​2.7, p-value ​= ​0.011), and from 12 to 26 weeks (mean change ​= ​3.5, p-value ​= ​0.009). Between the other two visits, there were no significant change in TMT-A t-score (Table 3). TMT-B t-score improved significantly from baseline to 6 weeks only (mean change ​= ​3.7, p-value ​= ​0.001) (Table 3). Symbol coding t-scores only had a significant improvement from baseline to 26 weeks (mean change ​= ​3.3, p-value ​= ​0.021) (Table 3). None of the cytokines changed significantly from baseline to end point in a linear mixed effects model (Fig. 2 and Table A2).

**Table 3:** Change in tests for psychomotor speed (t-score).

	TMT-A			TMT-B			SC		
	Estimates	SE	P	Estimates	SE	p	Estimates	SE	p
Baseline (0 weeks)	43.3	1.1	0.000	41.5	1.1	0.000	35.1	1.2	0.000
Change 0–6 weeks	2.7	1.1	**0.011**	3.7	1.1	**0.001**	1.9	1.1	0.084
Change 6–12 weeks	1.1	1.1	0.337	1.4	1.2	0.224	–	–	–
Change 6–26 weeks	–	–	–	–	–	–	-0.5	1.3	0.704
Change 12–26 weeks	3.5	1.3	**0.009**	2.5	1.3	0.064	–	–	–
Change 26–52 weeks	-1.0	1.4	0.499	2.8	1.5	0.055	3.3	1.4	**0.021**

Estimates from the linear mixed effects models. No measurements of SC at week 12.

Abbreviations: SC; symbol coding, TMT; Trail Making Test.

### 3.2 Relationship between psychomotor speed and cytokines

At baseline and end point, no Pearson's correlation coefficients reached the level of significance for the relationship between cytokines and any of the test TMT-A, TMT-B, or symbol coding (Table A3). In linear mixed effects models including measurements from all visits and correcting for possible confounders, only IFN-γ had a significant negative effect upon TMT-A (model estimate ​= ​-2.923, p ​= ​0.011) and symbol coding (model estimate ​= ​-2.564, p ​= ​0.038). None of the other cytokines had a significant effect upon TMT-A or symbol coding (Table 4). For TMT-B, we found no significant associations with the tested cytokines in a linear mixed effects model (Table 4).

**Table 4:** Estimated effect of log-transformed cytokines on t-scores from test of psychomotor speed.

	TMT-A			TMT-B			Symbol coding		
	Estimates	SE	*p*	Estimates	SE	*p*	Estimates	SE	*p*
IFN-γ	**-2.923**	**1.14**	**0.011**	-1.131	1.07	0.294	**-2.564**	**1.22**	**0.038**
IL-1β	0.161	0.55	0.768	-0.199	0.55	0.716	0.444	0.60	0.463
IL-10	0.835	0.58	0.149	0.550	0.56	0.330	-0.039	0.59	0.948
IL-12 p70	0.959	1.27	0.451	-0.867	1.26	0.493	1.044	1.31	0.427
IL-17a	-0.264	0.96	0.782	0.849	0.94	0.370	-1.602	1.15	0.166
IL-2	0.951	1.30	0.465	-0.607	1.27	0.635	0.248	1.346	0.854
IL-4	0.648	0.77	0.399	1.297	0.73	0.079	0.668	0.92	0.468
IL-6	-0.327	0.61	0.594	0.220	0.61	0.719	-0.576	0.68	0.399
TNF-α	-0.007	0.83	0.993	-0.217	0.83	0.793	1.516	0.92	0.103

Estimates from linear mixed effect models. The models included age, gender, BMI, ethnicity, smoking, study site, antipsychotic drug and PANSS positive score as independent variables.

Abbreviations: IFN; interferon, IL; interleukin, TMT; trail making test, TNF; transforming growth factor.

In linear mixed effects models excluding patients with F22 and F23 ICD-10 diagnoses, IFN-γ was still significantly associated with TMT-A (model estimate ​= ​-3.059, p ​= ​0.042), but not symbol coding (model estimate ​= ​-1.882, p ​= ​0.212). In addition, IL-4 was associated with TMT-A (model estimate ​= ​2.506, p ​= ​0.030) and TMT-B (model estimate ​= ​2.417, p ​= ​0.046). None of the other tested cytokines were significantly related to TMT-A or TMT-B (Table A4). In these linear mixed effects models, no cytokines had a significant effect upon symbol coding (Table A4). As in the main analyses described above, these models were also conducted including several possible confounders for correction.

## 4 Discussion

Our findings indicate that higher levels of IFN-γ had a significant negative effect upon psychomotor speed (assessed by TMT-A and symbol coding) in patients with a spectrum of psychotic disorders. No other cytokines were significantly associated with psychomotor speed. In addition, we found that although patients improved significantly on tests for psychomotor speed during the study period of 52 weeks, the tested cytokines remained stable without any significant changes.

To our knowledge, this was the first study to include IFN-γ when investigating the association between inflammatory markers and psychomotor speed in patients with severe mental disorders. Therefore, it is difficult to find studies that are directly comparable with the finding regarding IFN-γ and psychomotor speed in our study. However, previous studies have found an association between performance on tests for psychomotor speed (including TMT-A, TMT-B and symbol coding) and other pro-inflammatory cytokines in schizophrenia patients (Frydecka et al., 2015; Goldsmith et al., 2020). Additionally, Bulzacka et al. found that schizophrenia patients with increased levels of high-sensitivity C-reactive protein (hs-CRP, increased level defined as ​> ​3 ​mg/ml) performed significantly worse on TMT-A and TMT-B when compared to patients with normal hs-CRP levels (Bulzacka et al., 2016). The same association between worse performance on TMT-A and hs-CRP have been proven in patients with both bipolar disorder and major depressive disorder (Dickerson et al., 2013; Krogh et al., 2014). CRP is known to induce the production of IFN-γ (Van Vré et al., 2008). Therefore, findings of CRP and IFN-γ in relation to psychomotor speed, might be an expression of the same immunological mechanisms. For future research, CRP should be included.

Of the three tests for psychomotor speed, only TMT-A and symbol coding were significantly associated with a cytokine. Although all three tests included in this study measure psychomotor speed to some extent, they differentiate in whether they assess other cognitive domains as well. More specifically, TMT-A tests psychomotor speed and visuospatial abilities, whereas TMT-B also draws on higher cognitive abilities including working memory (Bowie and Harvey, 2006; Crowe, 1998). The symbol coding test measures psychomotor speed and attention (Keefe et al., 2008). In other words, TMT-A could be classified as the purest measure of psychomotor speed of the three. Keeping the differences of the tests in mind, our finding might indicate that IFN-γ is related to psychomotor speed and attention. In the future, including more tests that measure psychomotor speed more solely would be helpful to differentiate this association further (Hubel et al., 2013; Skogan et al., 2018).

After sensitivity analyses excluding patients with delusional disorder and acute transient psychosis, IFN-γ was still associated with TMT-A, but not symbol coding. In these analyses we lost some statistical power, and this might explain the loss of a significant association between IFN-γ and symbol coding. In addition, we found an association between IL-4 and the two tests TMT-A and TMT-B. Although false positive findings are more probable when performing additional statistical tests, the spectrum of schizophrenia patients might represent a different entity. Regarding blood levels of cytokines, a meta-analysis concluded that certain cytokines are trait markers (Miller et al., 2011), and that not all cytokines normalize during treatment (Tourjman et al., 2013).

In general, IL-4 represents a different part of the immune system than IFN-γ (Zhu et al., 2010) Whereas IFN-γ is important for type 1 ​T helper (th1) cells (Li et al., 2014), IL-4 is classified as part of the th2 response (Zhu et al., 2010). In our study, the association between IL-4 and IFN-γ is inverse, which makes sense considering the underlying biological function of these cytokines. More specifically, IFN-γ had a negative effect on psychomotor speed, whereas IL-4 had a positive effect. To our knowledge, no previous studies investigating the association between cytokines and psychomotor speed in psychosis included IL-4 or other th2 cytokines. However, a few previous studies found increased levels of th2 cytokines in schizophrenia (Borovcanin et al., 2012; Guo et al., 2015), although the results are conflicting (Momtazmanesh et al., 2019).

The majority of tested cytokines were not significantly associated with psychomotor speed in our study. This finding was somewhat surprising as it is contrary to a few previous studies which found an association between several cytokines and psychomotor speed in schizophrenia patients (Frydecka et al., 2015; Goldsmith et al., 2020). Still, our findings are in line with one study which also failed to find any significant correlation between plasma levels of the two cytokines IL-6 and TNF-α, and psychomotor speed in patients with schizophrenia (Hori et al., 2017). Similarly, no association between cytokines (IL-6, IL-10 and TNF-α) and psychomotor speed was found in a group of patients with bipolar disorder (Mora et al., 2019). In a longitudinal study by Krogh et al. including patients with major depressive disorder, only hs-CRP predicted an improvement on TMT-A, but not the cytokine IL-6 (Krogh et al., 2014). However, as mentioned above, none of these studies included IFN-γ or IL-4 in their analyses, making it difficult to meaningfully compare our results. Finally, differences between our and other studies regarding selection and exclusion criteria (i.e. diagnoses, somatic comorbidity, and substance abuse) might also explain different findings.

Interferons are potent cytokines with a major antiviral function (Pestka et al., 2004). IFN-α is commonly used as treatment for chronic hepatitis C together with an antiviral drug (AASLD-IDSA., 2021). When given IFN-α, patients do develop decreased psychomotor speed and depressive symptoms compared to controls (Majer et al., 2008). Another study has demonstrated that patients developing depression after IFN-α treatment have more reduced psychomotor speed when compared to somatically healthy patients with major depression (Capuron et al., 2009). Although IFN-γ and IFN-α are two different classes of interferons (class I and II), they have several similarities and these studies on IFN-α and psychomotor speed are therefore of relevance (Pestka et al., 2004).

On a neurobiological level, reduced psychomotor speed is associated with reduced white matter integrity in several parts of the brain, such as corpus callosum and basal ganglia (Karbasforoushan et al., 2015; Tsapanou et al., 2019; Wang et al., 2020). Such reduced white matter integrity may be induced by inflammation and peripheral cytokines (Benedetti et al., 2016; Najjar and Pearlman, 2015). In a study including patients with bipolar disorder, IFN-γ was associated with reduced white matter integrity in several networks of the brain (Benedetti et al., 2016). Further, IFN-γ is damaging to brain myelin through several mechanisms (Popko and Baerwald, 1999). Most importantly, through inducing apoptosis in oligodendrocytes (Buntinx et al., 2004; Horiuchi et al., 2006). In addition, activation of macrophages and microglia are of significance (Popko and Baerwald, 1999). These effects of IFN-γ on myelin are of importance in the pathology of the demyelinating disease multiple sclerosis (MS) (Kebir et al., 2009). Interestingly, reduction in psychomotor speed (measured by symbol coding and TMT-A) are the most common cognitive impairments in patients with MS (Grzegorski and Losy, 2017; Storm Van's Gravesande et al., 2019; Van Schependom et al., 2015).

When considering the pathophysiological mechanisms behind altered psychomotor activity, the basal ganglia need to be mentioned. In patients with schizophrenia, Yang et al. found an association between psychomotor slowing and basal ganglia activity (Yang et al., 2004). Following administration of IFN-α, hepatitis C patients do develop psychomotor slowing on neuropsychological tests, which is associated with reduced connectivity in the basal ganglia (Felger et al., 2016). An inflammatory stimulus (i.e. IFN-α or endotoxin) can influence the basal ganglia not only through reduced connectivity, but also alter dopamine metabolism and glutamate levels (Capuron et al., 2012; Eisenberger et al., 2010; Haroon et al., 2015). In fact, this relation between basal ganglia and inflammation might also involve reduced white matter integrity (Benedetti et al., 2016). In other words, it is possible that several pathophysiological mechanisms are important when understanding the link between psychomotor speed and inflammation.

In the current study, cytokines remained stable throughout the entire observational period of 52 weeks. Several studies have shown that some cytokines change from acute states of schizophrenia to more chronic phases (Çakici et al., 2021; Goldsmith et al., 2016a; Momtazmanesh et al., 2019). Still, certain cytokines remain unchanged during the course of schizophrenia, indicating that these cytokines are trait markers (Capuzzi et al., 2017; Tourjman et al., 2013). Importantly, patients from our study were in both acute and more chronic phases of schizophrenia. We did execute linear mixed effects models within the group of drug naïve patients, but here no cytokines affected the performance on psychomotor speed tests significantly (data not shown). The lack of an association between IFN-γ and TMT-A in the drug naïve group, could be due to low statistical power. Also, we did not distinguish between treatment groups, or responder's vs non-responders in our analyses. These are factors known to be of relevance when considering changes in cytokines over time in schizophrenia patients (Momtazmanesh et al., 2019; Mondelli et al., 2015; Tourjman et al., 2013).

In contrast to the cytokines remaining stable, patients improved significantly on tests for psychomotor speed during the study period. This in line with other studies demonstrating that schizophrenia patients treated with antipsychotics improve on overall cognitive performance (Fathian et al., 2019; Gold et al., 1999), and psychomotor speed over time (Hughes et al., 2003; Townsend et al., 2002). In this particular study population, we have found the same improvement in overall cognitive performance (Anda et al., 2021).

There are several limitations that need to be considered in this study. Only IFN-γ was significantly associated with psychomotor speed in the main analyses, and it is largely unclear why none of the other cytokines were associated with psychomotor speed. Considering a few previous studies on the topic, we expected to find an association between psychomotor speed and several inflammatory cytokines. However, as all tests for psychomotor speed changed significantly during the study period, it should have been possible to detect a relation with cytokines if present. The study was not powered for testing associations between cytokines and psychomotor speed and the lack of associations for other cytokines than IFN-γ may be a type II error. Even though correction for treatment groups did not have any effect on the main findings, we do not know from this study if antipsychotics in general influence the association between psychomotor speed and cytokines.

However, the study also has several strengths. Importantly, we used a hypothesis driven approach. An extensive panel of cytokines representing different immune responses were included. Regarding psychomotor speed, we included three validated and well-established neuropsychological tests. Although none of these tests are the gold standard for psychomotor speed, they are all considered sound measures of psychomotor speed. When measuring neuropsychological tests repeatedly, improved scores can be a result of practice effects. However, the tests included in this study is proven relatively stable to such effects (Rodriguez-Toscano et al., 2020). As already mentioned, TMT-A is the purest measure of psychomotor speed of the three, supporting that IFN-γ is associated with psychomotor speed specifically. Further, a longitudinal study design including a relatively large study sample, are also important strengths. Although being a RCT, the study was designed with few exclusion criteria, resembling a real-life sample of patients with psychotic disorders. Finally, we corrected for several potential confounders such as age, gender, BMI and severity of psychotic symptoms.

In conclusions, our findings suggest an association between IFN-γ and psychomotor speed over time. Future studies should elaborate this association further, including both a wide range of inflammatory markers and several tests for psychomotor speed.

## Role of funding source

This work was publicly funded entirely by 10.13039/501100005416The Research Council of Norway (grant number 213727), the 10.13039/501100004257Western Norway Regional Health Trust (grant number 911679) and the participating not-for-profit hospitals and universities. The funding sources had no role in study design, data collection, analysis and interpretation of data, writing of the paper or in the decision to publish.

## Declaration of competing interest

None.

## Footnotes

Appendix A Supplementary data to this article can be found online at https://doi.org/10.1016/j.bbih.2021.100392.
