# Supplementary material for: The association between cytokines and psychomotor speed in a spectrum of psychotic disorders: A longitudinal study
Source: Brain Behav Immun Health. 2021 Nov 23;18:100392. doi: 10.1016/j.bbih.2021.100392 (PMC8633579; doi:10.1016/j.bbih.2021.100392)
Supplement: Multimedia component 1 [file mmc1.docx]

**Appendices**

**Table A1: Coefficients of variation for serum cytokines**

|  | Intra-assay CVs | Inter-assay CVs |
| --- | --- | --- |
| IFN-γ | 10.6 | 9.3 |
| IL-1β | 10.5 | 13.6 |
| IL-10 | 10.0 | 4.7 |
| IL-12 p70 | 9.7 | 5.9 |
| IL-17a | 10.6 | 3.7 |
| IL-2 | 10.0 | 12.9 |
| IL-4 | 10.1 | 5.2 |
| IL-6 | 10.7 | 11.7 |
| TNF-α | 9.6 | 6.0 |

Abbreviations: CV; coefficients of variation, IFN; interferon, IL; interleukin, TNF; transforming growth factor

**Table A2: Change in log-transformed cytokines from baseline to end point**

|  | Estimates | SE | p-value |
| --- | --- | --- | --- |
| IFN-γ | 0.042 | 0.09 | 0.649 |
| IL-1β | -0.133 | 0.16 | 0.398 |
| IL-10 | -0.052 | 0.14 | 0.701 |
| IL-12 p70 | -0.070 | 0.07 | 0.289 |
| IL-17a | -0.058 | -0.11 | 0.597 |
| IL-2 | 0.145 | 0.09 | 0.098 |
| IL-4 | -0.001 | 0.09 | 0.988 |
| IL-6 | -0.024 | 0.14 | 0.866 |
| TNF-α | 0.075 | 0.10 | 0.448 |

Data estimated in linear mixed effect models

Abbreviations: IFN; interferon, IL; interleukin, TNF; transforming growth factor

**Table A3: Correlation coefficients (r) between tests for psychomotor speed and cytokines**

|  | TMT-A baseline (n = 98) | | TMT-A end point (n =42) | | TMT-B baseline (n = 86) | | TMT-B end point (n = 40) | | SC baseline  (n = 90) | | SC end point (n = 41) | |
| --- | --- | --- | --- | --- | --- | --- | --- | --- | --- | --- | --- | --- |
|  | r | *p* | r | *p* | r | *p* | r | *p* | r | *p* | r | *p* |
| IFN-γ | -0.139 | 0.174 | -0.188 | 0.234 | 0.029 | 0.793 | -0.208 | 0.197 | -0.152 | 0.153 | -0.134 | 0.403 |
| IL-1β^a^ | -0.016 | 0.889 | -0.053 | 0.745 | 0.106 | 0.378 | -0.177 | 0.288 | 0.031 | 0.794 | 0.135 | 0.413 |
| IL-10 | 0.008 | 0.940 | 0.185 | 0.242 | -0.004 | 0.972 | 0.121 | 0.456 | -0.090 | 0.370 | 0.186 | 0.244 |
| IL-12 p70^a^ | -0.096 | 0.388 | -0.228 | 0.157 | 0.081 | 0.498 | -0.253 | 0.125 | -0.074 | 0.530 | -0.078 | 0.636 |
| IL-17a | -0.045 | 0.684 | -0.171 | 0.291 | 0.110 | 0.353 | -0.256 | 0.121 | 0.001 | 0.990 | -0.217 | 0.185 |
| IL-2 | -0.021 | 0.837 | -0.077 | 0.627 | 0.110 | 0.312 | -0.184 | 0.256 | -0.015 | 0.890 | -0.122 | 0.449 |
| IL-4 | -0.006 | 0.837 | -0.038 | 0.809 | 0.139 | 0.202 | -0.103 | 0.527 | 0.057 | 0.596 | -0.003 | 0.983 |
| IL-6 | -0.059 | 0.956 | -0.078 | 0.625 | 0.129 | 0.237 | -0.163 | 0.317 | -0.037 | 0.726 | -0.010 | 0.951 |
| TNF-α | -0.070 | 0.495 | -0.120 | 0.449 | 0.050 | 0.650 | -0.243 | 0.131 | -0.060 | 0.574 | -0.081 | 0.613 |

^a^24 missing at baseline, 5 missing at end point

^b^23 missing at baseline, 5 missing at end point

Abbreviations: IFN; interferon, IL; interleukin, SC; symbol coding, TMT; trail making test, TNF; tumor necrosis factor

**Table A4: Estimated effect of log-transformed cytokines on t-scores from test of psychomotor speed in patients with schizophrenia spectrum disorders**

|  | TMT-A | | | TMT-B | | | Symbol coding | | |
| --- | --- | --- | --- | --- | --- | --- | --- | --- | --- |
|  | Model estimate | SE | *p* | Model estimate | SE | *p* | Model estimate | SE | *p* |
| IFN-γ | **-3.059** | **1.48** | **0.042** | -1.303 | 1.536 | 0.399 | -1.882 | 1.493 | 0.212 |
| IL-1β | -0.077 | 0.68 | 0.9104 | -0.255 | 0.724 | 0.726 | 0.535 | 0.758 | 0.483 |
| IL-10 | 0.638 | 0.68 | 0.349 | 0.521 | 0.697 | 0.456 | -0.400 | 0.694 | 0.566 |
| IL-12 p70 | -0.255 | 1.47 | 0.863 | -1.495 | 1.588 | 0.349 | 0.446 | 1.467 | 0.762 |
| IL-17a | -0.451 | 0.95 | 0.636 | 0.782 | 0.997 | 0.435 | -1.610 | 1.165 | 0.172 |
| IL-2 | 1.172 | 1.29 | 0.364 | -0.094 | 1.357 | 0.945 | 1.113 | 1.323 | 0.403 |
| IL-4 | **2.506** | **1.14** | **0.030** | **2.417** | **1.193** | **0.046** | -0.056 | 1.208 | 0.963 |
| IL-6 | -0.132 | 0.65 | 0.838 | 0.350 | 0.699 | 0.617 | -0.350 | 0.725 | 0.631 |
| TNF-α | -0.594 | 0.91 | 0.513 | 0.385 | -0.846 | 0.385 | 1.874 | 1.00 | 0.065 |

Data estimated in linear mixed effect models. The models included age, gender, BMI, ethnicity, smoking, study site, antipsychotic drug and PANSS positive score as independent variables. Patients with ICD-10 diagnoses F20, F21, F25 and F28-29 were included. N = 58 at baseline, N = 27 at end-point.

Abbreviations: IFN; interferon, IL; interleukin, TMT; trail making test, TNF; transforming growth factor
